# Prognostic significance of fibrinogen levels in sepsis-associated acute kidney injury: unveiling a nonlinear relationship and clinical implications

**DOI:** 10.3389/fneph.2024.1398386

**Published:** 2024-11-06

**Authors:** Manqin Chen, Xinbin Chen, Huaxiang Ling, Chengwen Bai, Lihua Chen, Lin Zhong, Ping Gong, Fei Shi

**Affiliations:** ^1^ Department of Emergency, Shenzhen People’s Hospital (The Second Clinical Medical College, Jinan University; The First Affiliated Hospital, Southern University of Science and Technology), Shenzhen, Guangdong, China; ^2^ Zhuhai International Travel Healthcare Center, Port Clinic of Gongbei Customs District, Zhuhai, Guangdong, China; ^3^ Department of Respiratory and Critical Care Medicine, Affiliated Hospital of Guangdong Medical University, Zhanjiang, Guangdong, China; ^4^ Department of Infectious Diseases, Institute of Shenzhen Respiratory Diseases, Shenzhen People’s Hospital (The Second Clinical Medical College, Jinan University, The First Affiliated Hospital, Southern University of Science and Technology), Shenzhen, Guangdong, China

**Keywords:** fibrinogen, sepsis, acute kidney injury, sepsis-associated acute kidney injury, mortality, medical information mart for intensive care-IV

## Abstract

**Background:**

Fibrinogen plays a pivotal role in the inflammatory cascade and is intricately linked to the pathogenesis of sepsis. Nevertheless, its significance as a prognostic marker for sepsis-associated acute kidney injury (SA-AKI) remains uncertain. This study aimed to investigate the association between fibrinogen levels and 28-day mortality with sepsis-associated acute kidney injury.

**Method:**

The fibrinogen levels of patients admitted to the intensive care unit of Beth Israel Deaconess Medical Center between 2008 and 2019 were retrospectively assessed, and those diagnosed with SA-AKI were divided into low, middle and high fibrinogen level groups according to tertiles. Multivariate Cox proportional hazards model was used to assess the 28-day mortality risk of the SA-AKI patients.

**Results:**

A total of 3,479 patients with SA-AKI were included in the study. Fibrinogen demonstrated an independent association with 28-day mortality, yielding a hazard ratio (HR) of 0.961 (95% confidence interval [CI]: 0.923-0.999, *P* = 0.0471). Notably, a non-linear relationship between fibrinogen levels and 28-day mortality was observed, with the threshold observed at approximately 1.6 g/l. The effect sizes and corresponding CIs below and above this threshold were 0.509 (0.367, 0.707) and 1.011 (0.961, 1.064), respectively. Specifically, the risk of mortality among SA-AKI patients decreased by 49.1% for every 1 g/l increment in fibrinogen, provided that fibrinogen levels were less than 1.6 g/l.

**Conclusion:**

In patients with SA-AKI, a non-linear relationship was identified between fibrinogen levels and 28-day mortality. Particularly, when their fibrinogen levels were less than 1.6 g/l, a concomitant decrease in 28-day mortality was observed as fibrinogen levels increased.

## 1 Introduction

Acute kidney injury is defined as the abrupt deterioration of kidney function, characterized by elevated levels of creatinine and decreased urine output (1, 2). In the occurrence and progression of AKI, inflammation plays a crucial role (3, 4). Disruption of the inflammation-immune response is at the core of sepsis development (5), which can severely impair kidney function. The kidney is an important target organ in sepsis. If sepsis patients are not promptly treated, they are susceptible to deteriorating into sepsis-associated acute kidney injury within seven days (6, 7), accounting for approximately 45-70% of all critically ill patients (8–10). Furthermore, SA-AKI is closely associated with an increased risk of in-hospital mortality. Bagshaw et al. (11) reported that the in-hospital mortality rate of SA-AKI patients was 1.48 times higher than that of non-septic AKI patients, with a significantly longer average length of hospital stay for SA-AKI (37 days *vs* 21 days). Another study indicated that SA-AKI patients had a significantly higher in-hospital mortality rate compared to non-septic AKI patients (64.81% *vs* 22.22%) (12). Although many studies have revealed significant differences between SA-AKI and non-septic AKI, the prognostic indicators for SA-AKI still remain uncertain.

Fibrinogen, a precursor synthesized in the liver, plays a dual role as a clotting factor and an acute-phase inflammatory marker. It occurs actively during the period of sepsis and is associated with inflammatory response, consumptive coagulopathy, and microthrombus formation (13). Recent research reports a correlation between fibrinogen and prognosis in various clinical contexts, including tumor outcomes, cardiovascular surgery, and contrast-induced AKI (14–17). Moreover, propensity score matching studies have demonstrated a significant association between elevated fibrinogen levels and reduced risk of death in sepsis patients (HR 0.66, *P* < 0.001 and HR 0.73, *P* < 0.001) (18). Previous research shows that fibrinogen has different implications for AKI in different diseases. For instance, in liver transplant recipients, low levels of fibrinogen serve as a risk factor for the development of acute kidney injury (AKI) (19). Conversely, in patients undergoing heart valve replacement surgery, high fibrinogen levels are considered a risk factor for postoperative AKI (15). Additionally, there is a controversial relationship between fibrinogen and prognosis in sepsis as depicted by different literature sources. Studies based on Japanese databases demonstrate a J-shaped relationship, while the Mimic database shows a linear correlation (18, 20). In severe AKI cases, a linear relationship is observed (21). However, the significance of fibrinogen in the context of SA-AKI remains largely unknown due to the absence of relevant data. Therefore, the main objective of our study is to evaluate the predictive value of fibrinogen for the 28-day mortality in SA-AKI patients.

## 2 Materials and methods

### 2.1 Data source

This is a retrospective observational cohort study based on data retrieved from the Medical Information Mart for Intensive Care-IV (MIMIC-IV) 2.0 database, which is managed by the Beth Israel Deaconess Medical Center. The study included over 50,000 first-time intensive care unit (ICU) admissions for sepsis between 2008 and 2019 (22). Author Chen successfully completed the training on “Protection of Human Subjects” and obtained authorization to access this database (certification number 51474639). Patient identifiers and data information were de-identified. The institutional review boards of Massachusetts Institute of Technology and Beth Israel Deaconess Medical Center approved the study protocol and use of the database for research purposes.

### 2.2 Study population

**Figure 1:**
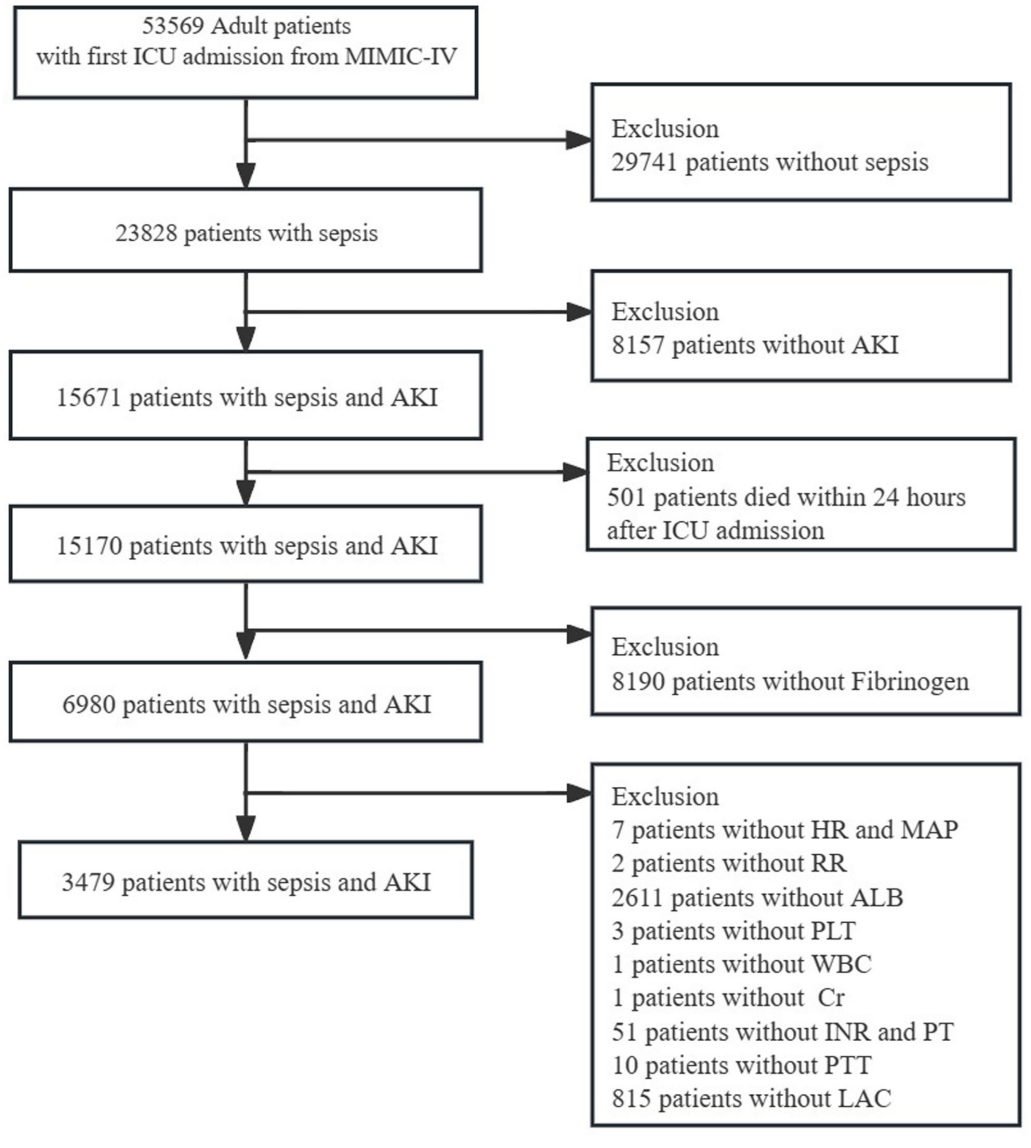
Flowchart of patient selection. MIMIC-IV, Medical Information Mart for Intensive Care-IV; AKI, Acute kidney injury; HR, Heart Rate; MAP, mean arterial pressure; RR, respiratory rate; ALB, albumin; WBC, white blood cell; Cr, creatinine; INR, international normalized ratio; PT, prolonged prothrombin time; PTT, partial thromboplastin time; LAC, lactate.

Data on individuals from the MIMIC-IV database who were diagnosed with sepsis according to the Sepsis-3 criteria (6) were retrieved. The KDIGO served as the basis for defining AKI (7). In this study, sepsis patients diagnosed with AKI within 2 days of admission to the ICU were selected as SA-AKI. The study inclusion criteria were:(1) only the first ICU admission was considered, and corresponding data were recorded; (2) patients aged ≥ 18 years old; (3) their length of ICU stays exceeded 24 hours; (4) data on important parameters such as fibrinogen levels and vital signs were completely recorded. Exclusion criteria:(1) Patients die in the first 24 h; (2) Patients without incomplete datasets. The final cohort comprised 3,479 participants (**Figure 1**). This study was approved by the Ethics Committee of Shenzhen People’s Hospital (Ethics number: LL-KT-201705001).

### 2.3 Main exposure

We used the Structured Query Language (SQL) to obtain fibrinogen data within the initial 24 hours of ICU admission. In cases where multiple measurements were available, we considered the lowest recorded fibrinogen level. Tertiles were used to classify the fibrinogens into the following three groups: low (fibrinogen ranging from 0.26 to 1.68 g/l), middle (fibrinogen ranging from 1.69 to 3.22 g/l), and high (fibrinogen ranging from 3.23 to 14.66 g/l).

### 2.4 Covariates and measurements

On the basis of previous reports (18, 21, 23), the following variables were included to confirm the association between outcomes and fibrinogen: demographic characteristics such as age and gender, vital signs and laboratory tests including heart rate (HR), mean arterial pressure (MAP), respiratory rate, white blood cell count (WBC), serum albumin (ALB), platelet count (PLT), serum creatinine (Cr), international normalized ratio (INR), partial thromboplastin time (PTT), prothrombin time (PT), and lactate (LAC), as well as comorbidities such as myocardial infarction (MI), congestive heart failure (CHF), cerebrovascular disease, chronic obstructive pulmonary disease (COPD), diabetes (DM), chronic kidney disease (CKD), cancer, liver disease, and metastatic solid tumor (MST). Additionally, we considered severity assessment scores, including the Sequential Organ Failure Assessment (SOFA) score, Simplified Acute Physiology Score III (SAPS III), and the Charlson Comorbidity Index (CCI). Furthermore, we took into account treatments administered on the first day, encompassing mechanical ventilation (MV), renal replacement therapy (RRT), and norepinephrine (NE). Vital signs are represented by mean values, while laboratory test results are based on the most adverse values recorded on the initial day of ICU admission.

Sepsis‐induced coagulopathy (SIC) was diagnosed using International Society on Thrombosis and Hemostasis (ISTH)scoring systems (24). CKD was divided into 5 stages according to glomerular filtration rate (GFR). The GFR is estimated from Chronic Kidney Disease Epidemiology Collaboration (CKD- EPI) as follow (25):

GFR=a x (Cr/b/88.4)^^c^ x (0.993)^^age^

a: female =144, male=141.

b: female =0.7, male =0.9.

c: female: Cr ≤ 61.88 umol/l, c=-0.329; Cr >61.88 umol/l, c=1.209.

male: Cr ≤ 61.88 umol/l,c =-0.411; Cr >61.88 umol/l, c =-1.20.

### 2.5 Primary results

The primary outcome was 28-day mortality.

### 2.6 Statistical analysis

Descriptive analyses based on the fibrinogen tertials were performed. Categorical variables are presented using frequencies and percentages, and continuous variables are expressed as means with standard deviations (SD) for normally distributed data or medians with interquartile ranges (IQR) for skewed distributions. For statistical comparisons, we utilized the chi-square test for categorical variables, the one-way analysis of variance (ANOVA) for normally distributed data, and the Kruskal-Wallis test for skewed data. Survival analysis was performed using Kaplan-Meier curves and log-rank tests.

Univariate and multivariable Cox regression analyses were conducted to assess the relationship between fibrinogen levels and 28-day mortality. Confounding factors were addressed using multivariable-adjusted models. Model 1 adjusted for age and gender. Model 2 included adjustments from Model 1 and additional variables such as MAP, respiratory rate, HR, PLT, WBC, Cr, PT, PTT, INR, ALB and LAC. Model 3 included adjustments from Model 2 and additional variables such as MI, CHF, cerebrovascular disease, COPD, DM, CKD, cancer, liver disease, MST, SOFA, APS III, CCI, MV, RRT and NE.

We used a generalized additive model (GAM) to identify non-linear relationships. A two-piecewise linear regression model was used to assess the threshold effect of the fibrinogen and SA-AKI according to the smoothed curves. In order to ascertain the optimal threshold levels of fibrinogen, a recursive approach was employed, with the maximum model likelihood selected as the optimal outcome. A comparison was made between a one-line linear regression model and a two-piecewise linear model using a likelihood ratio test. Subgroup assessments were performed for sex, age, MAP, INR, PTT, PT, LAC, SOFA, COPD, DM, CKD, liver disease, MV, RRT and NE using a stratified Cox regression model. Likelihood ratio tests were employed to examine the interactions between subgroups. A sensitivity analysis was conducted to evaluate the relationship between fibrinogen and 28-day mortality in individuals with liver disease and SIC. Patients with SA-AKI with missing covariates were deleted, and baseline characteristics were compared between the missing and non-missing groups to assess the effect of missing data on outcomes.

All statistical analyses were carried out using R 3.3.2 from the R Foundation (http://www.R-project.org) and Free Statistics version 1.7. Statistical significance was determined using a two-sided test with a significance level of *P* < 0.05.

## 3 Results

### 3.1 Participants selection

Among the 53,569 admissions reported in MIMIC-IV, a total of 15,671 patients with SA-AKI were identified. The study protocol is schematically represented in **Figure 1**. Based on the inclusion and exclusion criteria, a total of 3,479 patients with comprehensive data were included for analysis.

### 3.2 Demographics and baseline characteristics

**Table 1** presents the baseline characteristics of the participants, categorized into groups based on fibrinogen tertials: the low group (≥0.26 to ≤1.68 g/l), the middle group (≥1.69 to ≤3.22 g/l), and the high group (≥3.23 to ≤14.66 g/l). The average age of all participants was 62.5 ± 15.8 years, with 60.3% being male. In the low group, patients were younger and presented more severe conditions, as indicated by lower PLT, higher LAC levels, INR, PT, PTT, SOFA score and SAPS III. These patients also required more life support measures, including a higher utilization of MV and RRT. Conversely, the high group exhibited a higher prevalence of chronic comorbidities such as MI, CHF, COPD, DM, CKD, MST, and CCI. **Supplementary Table S5** shows little differences in baseline characteristics between the missing and non-missing groups.

**Table 1:** Baseline and clinical characteristics of the study population according to Fibrinogen.

Variables	fibrinogen(g/l)				
	Total (n = 3479)	Low (0.26-1.68) (n = 1152)	Mid (1.69-3.22) (n = 1159)	High (3.23-14.66) (n = 1168)	*P* value
Baseline characteristics					
Sex(Male),n (%)	2098 (60.3)	670 (58.2)	716 (61.8)	712 (61)	0.176
Age(years)	62.5 ± 15.8	59.7 ± 15.7	64.0 ± 15.3	63.9 ± 15.9	< 0.001*
Vital signs					
HR (bpm)	90.8 ± 17.5	90.6 ± 16.8	89.2 ± 16.9	92.4 ± 18.7	< 0.001*
MAP (mmHg)	75.8 ± 9.7	75.4 ± 9.7	75.8 ± 9.8	76.3 ± 9.5	0.08
Respiratory-rate (bpm)	20.3 ± 4.5	19.8 ± 4.5	19.8 ± 4.4	21.3 ± 4.4	< 0.001*
Laboratory examination					
PLT(×10^9^/L)	119.0 (72.0, 179.0)	79.0 (50.0, 120.0)	125.0 (84.0, 175.0)	166.0 (109.0, 227.0)	< 0.001*
WBC(×109/L)	9.5 (6.0, 13.5)	8.3 (5.3, 12.2)	9.8 (6.6, 13.4)	10.4 (6.6, 15.1)	< 0.001*
ALB(g/l)	25.7 ± 6.2	25.2 ± 6.4	26.9 ± 6.4	24.9 ± 5.6	< 0.001*
Cr(mg/dL)	1.1 (0.8, 1.8)	1.1 (0.8, 1.7)	1.0 (0.7, 1.6)	1.2 (0.8, 2.1)	< 0.001*
INR	1.5 ± 0.7	1.6 ± 0.7	1.4 ± 0.6	1.4 ± 0.7	< 0.001*
PT(s)	16.3 ± 7.1	17.5 ± 7.7	15.6 ± 6.4	15.6 ± 7.1	< 0.001*
PTT(s)	33.5 ± 11.7	35.9 ± 12.9	32.1 ± 9.9	32.5 ± 11.7	< 0.001*
LAC (mmol/l)	1.6 (1.1, 2.4)	1.9 (1.3, 3.1)	1.5 (1.1, 2.2)	1.5 (1.1, 2.2)	< 0.001*
Disease severity score					
SOFA	4.0 (3.0, 6.0)	5.0 (3.0, 7.0)	4.0 (3.0, 6.0)	4.0 (3.0, 5.0)	< 0.001*
SAPS III	74.1 ± 30.3	79.1 ± 31.8	67.6 ± 30.0	75.5 ± 27.7	< 0.001*
Comorbidities					
MI (%)	678 (19.5)	153 (13.3)	266 (23)	259 (22.2)	< 0.001*
CHF (%)	1085 (31.2)	253 (22)	412 (35.5)	420 (36)	< 0.001*
Cerebrovascular-disease (%)	433 (12.4)	144 (12.5)	128 (11)	161 (13.8)	0.134
COPD (%)	884 (25.4)	234 (20.3)	328 (28.3)	322 (27.6)	< 0.001*
DM (%)	311 (8.9)	54 (4.7)	109 (9.4)	148 (12.7)	< 0.001*
CKD (%)	805 (23.1)	198 (17.2)	288 (24.8)	319 (27.3)	< 0.001*
Liver (%)	1049 (30.2)	555 (48.2)	318 (27.4)	176 (15.1)	< 0.001*
Cancer (%)	528 (15.2)	179 (15.5)	164 (14.2)	185 (15.8)	0.481
MST (%)	157 (4.5)	35 (3)	51 (4.4)	71 (6.1)	0.002*
CCI	6.0 ± 2.9	5.8 ± 2.7	6.2 ± 2.9	6.0 ± 3.0	0.01*
Treatments using on day 1					
MV (%)	2900 (83.4)	986 (85.6)	999 (86.2)	915 (78.3)	< 0.001*
RRT (%)	406 (11.7)	160 (13.9)	117 (10.1)	129 (11)	0.013*
NE (%)	1595 (45.8)	529 (45.9)	479 (41.3)	587 (50.3)	< 0.001*

*P < 0.05; HR, Heart Rate; MAP, mean arterial pressure; PLT, platelet; WBC, white blood cell; ALB, albumin; Cr, creatinine; INR, international normalized ratio; PTT, partial thromboplastin time; PT, prolonged prothrombin time; LAC, lactate; SOFA, sequential organ failure assessment; SAPS III, simplified acute physiology score III; MI, myocardial infarct; CHF, congestive heart failure; COPD, chronic obstructive pulmonary disease; DM, diabetes; CKD, chronic kidney disease; MST, metastatic solid tumor; CCI, charlson comorbidity index; MV, mechanic ventilation; RRT, renal replacement therapy; NE, norepinephrine.

### 3.3 Association between fibrinogen and prognosis of SA-AKI

**Figure 2:**
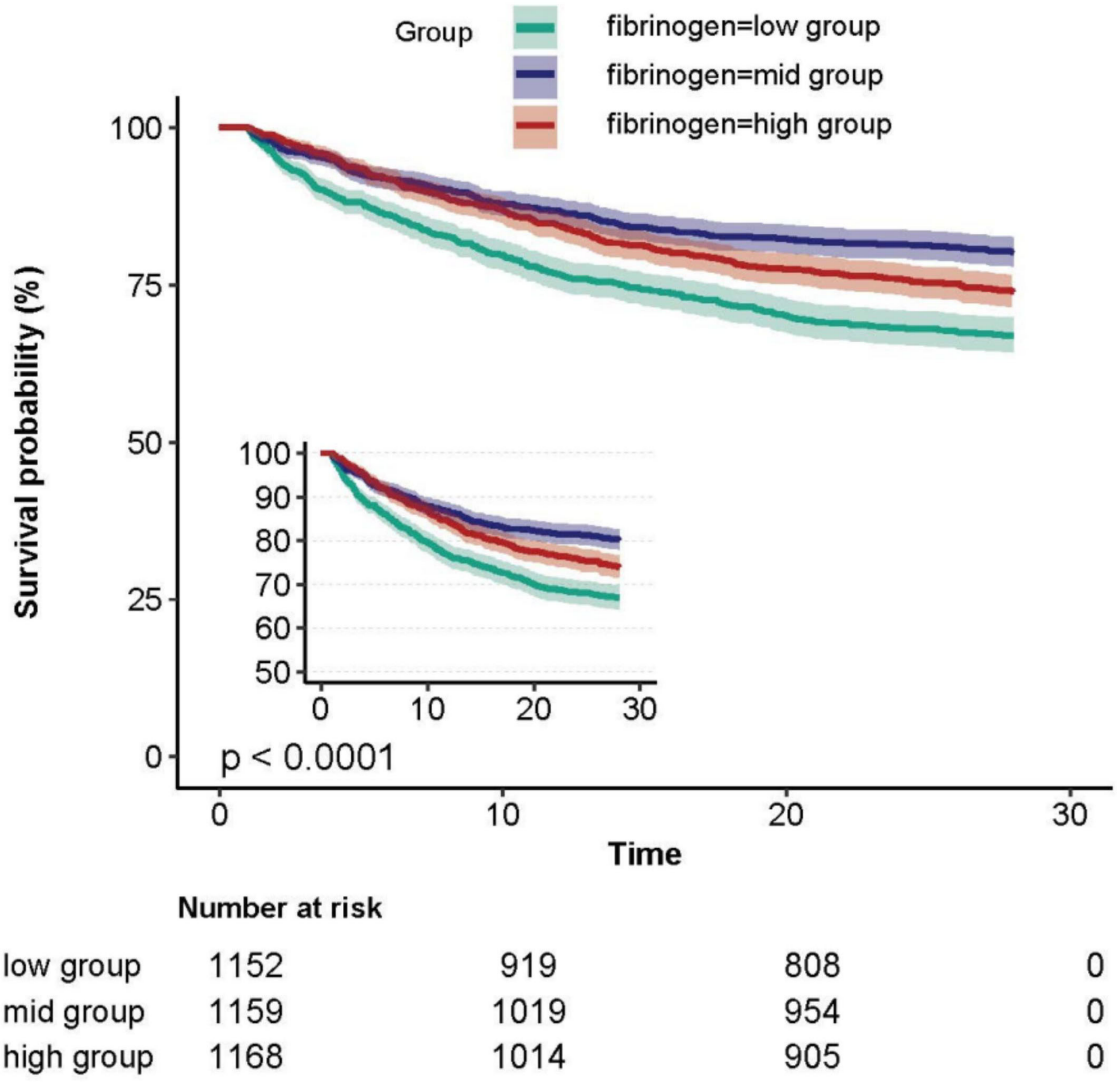
Kaplan-Meier Survival Curves for 28 daysof patients with SA-AKI depending on fibrinogen.

The Kaplan-Meier curve demonstrated that patients in the fibrinogen low group exhibited the highest 28-day mortality rate, while those in the middle group had the lowest mortality rate (**Figure 2**).

We examined the relationship between fibrinogen levels and 28-day mortality of SA-AKI using both univariate and multivariate Cox proportional hazards regression methods (**Table 2**). In the univariate analysis, several factors, including sex, age, MAP, respiratory rate, HR, fibrinogen, PLT, WBC, Cr, PT, PTT, INR, ALB, LAC, MI, CHF, CKD, cancer, liver disease, MST, SOFA, APS III, CCI, MV, RRT and NE showed significant association with 28-day mortality (*P*<0.001). However, on multivariate analysis, only age, respiratory rate, fibrinogen, WBC, PT, PTT, ALB, MI, liver disease, MST, APS III, MV and norepinephrine were identified as independent factors.

**Table 2:** Univariate Cox proportional hazards regression analysis the association between Fibrinogen and 28-day mortality in Patients With SA-AKI.

Variable	Univariate Cox proportional hazards regression HR ^1^ (95% CI)	*P* value	Multivariate Cox proportional hazards regression HR _1_ (95% CI)	*P* value
Sex (Male),n(%)	0.8 (0.7,0.91)	< 0.001*	0.88 (0.77~1.01)	0.061
Age (years)	1.01 (1.01,1.02)	< 0.001*	1.02 (1.01~1.03)	<0.001*
HR ^2^ (bpm)	1.01 (1.01,1.01)	< 0.001*	1 (1~1.01)	0.312
MAP (mmHg)	0.98 (0.97,0.99)	< 0.001*	1 (0.99~1.01)	0.574
Respiratory-rate (bpm)	1.07 (1.06,1.09)	< 0.001*	1.03 (1.01~1.05)	<0.001*
fibrinogen (g/l)	0.95 (0.92,0.98)	0.003*	0.961 (0.923~0.999)	0.047*
Low	1(reference)	–	1(reference)	–
Mid	0.55 (0.47,0.65)	< 0.001*	0.67 (0.56~0.8)	<0.001*
High	0.74 (0.63,0.86)	< 0.001*	0.81 (0.67~0.97)	0.024*
ALB(g/l)	0.96 (0.95,0.97)	< 0.001*	1.01 (1~1.03)	0.034*
PLT(×109/L)	0.9992 (0.9984,1)	0.043*	1 (1~1)	0.398
WBC(×109/L)	1.01 (1.01,1.01)	< 0.001*	1.01 (1~1.01)	<0.001*
Cr(mg/dL)	1.06 (1.02,1.1)	0.002*	0.97 (0.92~1.03)	0.322
INR	1.36 (1.3,1.43)	< 0.001*	1.16 (0.58~2.32)	0.673
PT(s)	1.03 (1.02,1.03)	< 0.001*	1 (0.94~1.06)	0.965
PTT(s)	1.02 (1.02,1.02)	< 0.001*	1.01 (1~1.01)	<0.001*
LAC (mmol/l)	1.23 (1.2,1.26)	< 0.001*	1.09 (1.06~1.13)	<0.001*
SOFA	1.1 (1.07,1.12)	< 0.001*	1.02 (0.99~1.04)	0.193
SAPS III	1.02 (1.02,1.02)	< 0.001*	1.02 (1.01~1.02)	<0.001*
MI	1.24 (1.06,1.45)	0.006*	1.23 (1.03~1.47)	0.019*
CHF	1.14 (0.99,1.3)	0.07	0.98 (0.84~1.15)	0.837
Cerebrovascular- disease	1.14 (0.95,1.38)	0.156	1.07 (0.88~1.31)	0.487
COPD	1.1 (0.95,1.27)	0.196	0.98 (0.83~1.16)	0.833
CKD	1.04 (0.89,1.21)	0.647	0.82 (0.67~1.01)	0.066
DM	0.9 (0.71,1.13)	0.359	0.85 (0.65~1.13)	0.271
Liver	1.74 (1.53,1.99)	< 0.001*	1.36 (1.14~1.63)	0.001*
Cancer	1.45 (1.23,1.71)	< 0.001*	0.91 (0.73~1.13)	0.385
MST	2.72 (2.18,3.4)	< 0.001*	2.05 (1.43~2.93)	<0.001*
CCI	1.13 (1.11,1.16)	< 0.001*	1.05 (1~1.11)	0.07*
MV	0.77 (0.66,0.91)	0.002*	0.83 (0.7~0.98)	0.029*
RRT	1.61 (1.35,1.92)	< 0.001*	0.95 (0.78~1.17)	0.656
NE	1.96 (1.71,2.23)	< 0.001*	1.19 (1.02~1.38)	0.023

*P < 0.05;HR ^1^hazard ratio;HR ^2^ *Heart Rate*; MAP, mean arterial pressure; ALB, albumin; PLT, platelet; WBC, white blood cell; Cr, creatinine; INR, international normalized ratio; PTT, partial thromboplastin time; PT, prolonged prothrombin time; LAC, lactate; SOFA, sequential organ failure assessment; SAPS III, simplified acute physiology score III; MI, myocardial infarct; CHF, congestive heart failure; COPD, chronic obstructive pulmonary disease; CKD, chronic kidney disease; DM, diabetes; MST, metastatic solid tumor; CCI, charlson comorbidity index; MV, mechanic ventilation; RRT, renal replacement therapy; NE, norepinephrine.

The association with fibrinogen as a continuous variable remained significant (HR, 0.961; 95% CI, 0.923-0.999; *P*=0.0471; **Table 3**), even after adjusting for all possible covariates (**Table 3**, model 3).

**Table 3:** Multivariable Cox proportional hazards regression models evaluating the association between Fibrinogen and 28-day mortality in patients with SA-AKI.

Variable	unadjusted		Model 1		Model 2		Model 3	
	HR(95%)	*P* value	HR(95%)	*P* value	HR(95%)	*P* value	HR(95%)	*P* value
fibrinogen(g/l)	0.95 (0.92~0.98)	0.003*	0.94 (0.91~0.98)	0.001*	0.94 (0.91~0.98)	0.002*	0.961(0.923~0.999)	0.0471*
fibrinogen(g/l)								
Low (0.26-1.68)	1(reference)	_	1(reference)	_	1(reference)	_	1(reference)	_
Mid (1.69-3.22)	0.55 (0.47~0.65)	<0.001*	0.52 (0.44~0.61)	<0.001*	0.61 (0.51~0.72)	<0.001*	0.67 (0.56~0.8)	<0.001*
High(3.23-14.66)	0.74 (0.63~0.86)	<0.001*	0.7 (0.6~0.81)	<0.001*	0.72 (0.61~0.86)	<0.001*	0.81 (0.67~0.97)	0.024*
*P* for trend	0.84 (0.78~0.91)	<0.001*	0.82 (0.76~0.89)	<0.001*	0.84 (0.77~0.92)	<0.001*	0.9 (0.82~0.99)	0.024*

*P < 0.05; HR, hazard ratio.

Model 1: sex, age.

Model 2: sex, age, MAP, Respiratory-rate, HR, PLT, WBC, Cr, PT, PTT, INR, ALB, LAC.

Model 3: sex, age、MAP、Respiratory-rate, HR、PLT, WBC, CR, PT, PTT, INR, ALB, LAC, MI, CHF, cerebrovascular-disease, COPD, DM, CKD; cancer, liver-disease, MST, SOFA, SAPS-III, CCI, MV, RRT, NE.

In the fully adjusted model, including fibrinogen as a categorical variable, the 28-day mortality risk was found to be 33% lower (HR, 0.67; 95% CI, 0.56-0.8; *P <*0.001) for patients in the middle fibrinogen group and 19% lower (HR, 0.81; 95% CI, 0.67-0.97; *P* =0.024) for those in the high fibrinogen group compared to patients in the low fibrinogen group. Trend analysis indicated a significant trend, with a *P* trend value of 0.024). All models showed consistent and strong statistical results (**Table 3**).

### 3.4 Threshold effect analysis of fibrinogen on mortality of sepsis with AKI

**Figure 3:**
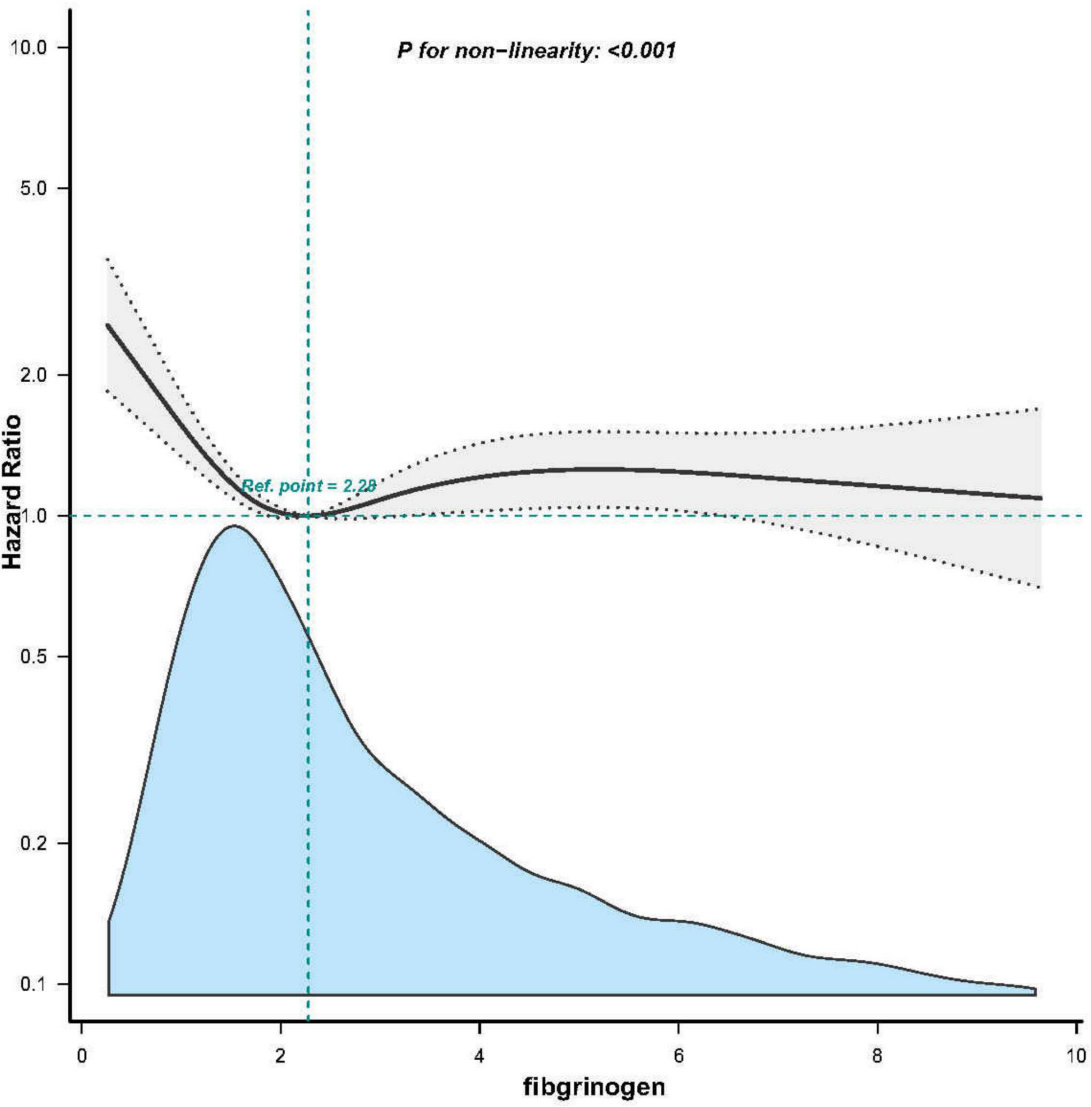
Associations between Fibrinogen concentrations with 28-day mortality among participants with SA-AKI in MIMIC-IV. HRs were adjusted for Model 3 and only 99.5% of the data is shows.

A non-linear dose-response relationship between fibrinogen and 28-day mortality was observed after adjusting for the indicated covariates (**Figure 3**). Using a two-piecewise linear regression model, we identified a threshold for fibrinogen at 1.6 g/l (**Table 4**). Below this threshold, there was a substantial decrease in 28-day mortality as fibrinogen levels increased (HR 0.509; 95% CI, 0.367-0.707; *P* < 0.001; **Table 4** and **Figure 2**). However, above the threshold, the estimated dose-response curve appeared to be relatively flat (HR 1.011; 95% CI, 0.961-1.064; *P* = 0.675; **Table 4**).

**Table 4:** The non-linearity relationship between Fibrinogen and 28-day mortality in patients with SA- AKI.

fibrinogen	HR (95%)	*P*-value
<1.6	0.509 (0.367,0.707)	<0.001
≥1.6	1.011 (0.961,1.064)	0.675
Likelihood Ratio test	–	<0.001

*P < 0.05; HR, hazard ratio.

Only 99.5% of the data is displayed.

### 3.5 Subgroup analyses and sensitivity analysis

**Figure 4:**
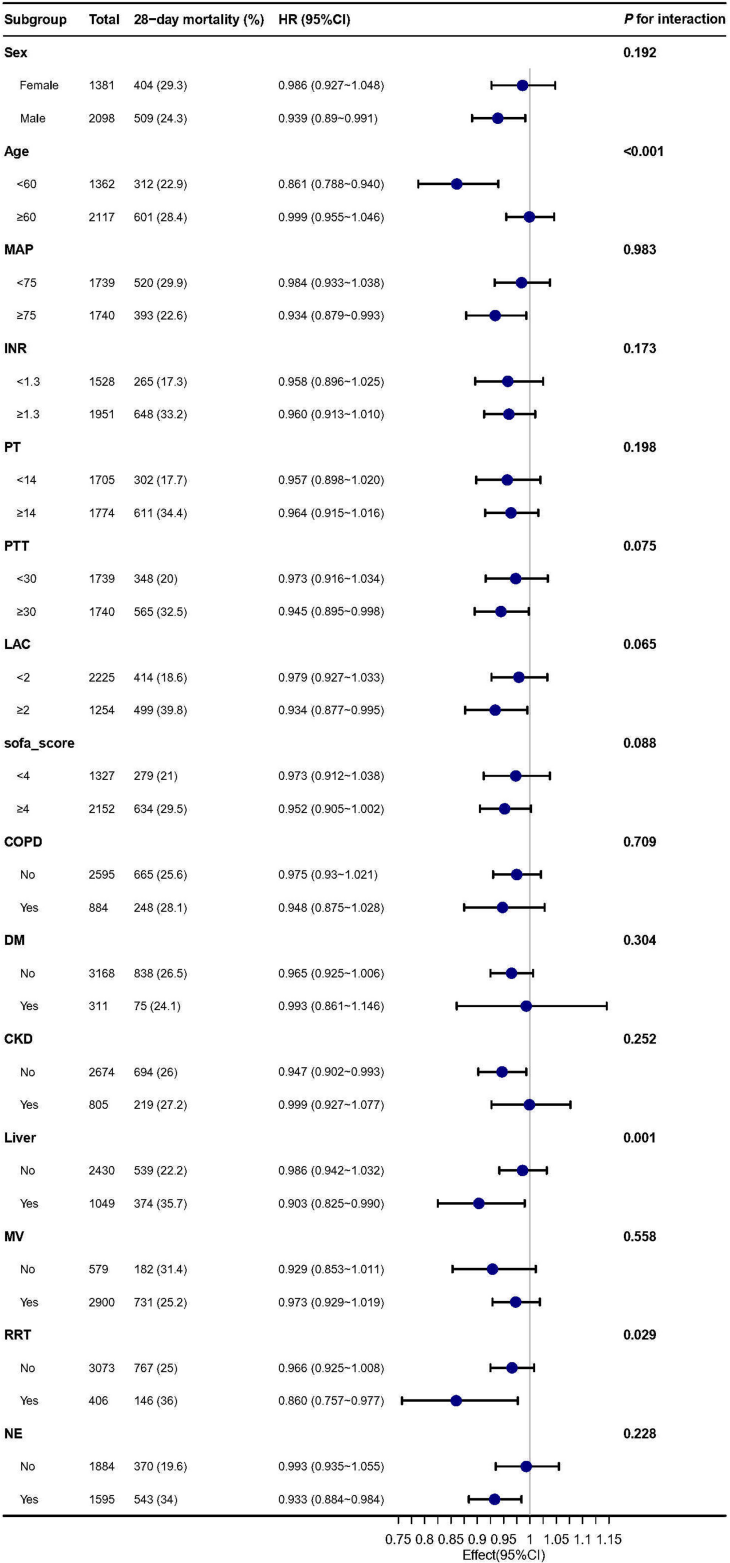
Subgroup analysis of the association between Fibrinogen and 28-day mortality in patients with sepsis and AKI. MAP, mean arterial pressure; INR, international normalized ratio; PT, prolonged prothrombin time; PTT, partial thromboplastin time; LAC, lactate; SOFA, sequential organ failure assessment; COPD, chronic obstructive pulmonary disease; DM, diabetes; CKD; MV, mechanic ventilation; RRT, renal replacement therapy; NE, norepinephrine.

The findings indicate a consistent and robust association between fibrinogen and 28-day mortality in sepsis patients with AKI, as demonstrated in **Figure 4**. Interactions were observed within specific subgroups, such as age, liver disease, and the use of renal replacement therapy (RRT) on day 1. we further divided the GFR into five categories, each representing different degrees of renal function (**Supplementary Table S1**). Subsequently, G 1 and G 2 were combined according to CKD risk stratification, while G 3, G 4, and G 5 were combined for stratification and interaction analysis. To assess the association of fibrinogen and SA-AKI with 28-day mortality outcomes in subgroups of GFR and CKD (**Supplementary Table S2**). After adjustment for all covariates, we observed similar results according to CKD (interaction *P* = 0.252) and GFR category subgroups (interaction *P* = 0.076). Furthermore, the sensitivity analysis conducted specifically on the liver disease and SIC populations provided reliable evidences regarding the relationship between fibrinogen and 28-day mortality, with an HR of 0.9 (95% CI: 0.82~0.99, *P* = 0.029) and HR of 0.97 (95% CI: 0.92~1.02, *P* = 0.214) (**Supplementary Tables S3**, **S4**).

## 4 Discussion

In this retrospective cohort study, we observed a non-linear relationship between fibrinogen and SA-AKI. Specifically, our findings indicate that when fibrinogen levels fell below 1.6 g/L, there was a pronounced reduction in mortality as fibrinogen levels increased. To the best of our knowledge, this is the first study elucidating such a non-linear association between fibrinogen and SA-AKI.

Previous studies have reported a positive association between elevated fibrinogen levels and an increased risk of developing AKI following cardiac valve replacement surgery (15). Fibrinogen, functioning as a coagulation factor, is known to elevate blood viscosity, potentially leading to renal hypoperfusion and hypoxia, thus contributing to the onset of AKI. Consequently, fibrinogen appears to exert a negative influence on both the development and outcomes of AKI. Nevertheless, a study focusing on living donor liver transplantation revealed that the occurrence of AKI was twice as frequent in the low fibrinogen group compared to the normal fibrinogen group (19). Further, our research reveals that individuals with SA-AKI and low fibrinogen levels had increased mortality rates compared to those with medium and high fibrinogen levels. These contrasting findings suggest that the influence of fibrinogen on AKI onset and progression may vary depending on the underlying disease etiology.

Sepsis emerges as the primary precipitating factor for AKI (5, 26). Fibrinogen’s role extends beyond conventional hemostasis in sepsis and plays a critical role in inflammatory responses, tissue injury, cellular migration, and gene expression (16). Research indicates a significant association between lower fibrinogen levels and increased mortality rates in sepsis (18, 20), and pediatric sepsis studies have also confirmed an inverse relationship between fibrinogen and in-hospital mortality rates (OR 0.767) (27). Fibrinogen, as a biomarker of coagulation factor consumption and an acute-phase reactant, rapidly increases during early-stage infections and inflammatory diseases, playing a crucial role in antimicrobial defense and hemostasis (28). Reports indicate sustained elevation of fibrinogen during the course of sepsis to counter inflammatory responses (29).Therefore, elevated fibrinogen levels early in sepsis may benefit host healing processes, while reduced levels could impair the ability to fight infection and regulate inflammation, thereby worsening sepsis prognosis (30). This study affirms the negative correlation (HR=0.961) between fibrinogen as a continuous variable and the 28-day mortality rate of SA-AKI. Compared to the low fibrinogen group, the medium and high fibrinogen groups experience respective reductions in the risk of mortality by 33% and 19%. Additionally, the low fibrinogen group demonstrates higher disease severity scores, including SOFA and APS III, indicating a more severe condition and a poorer prognosis.

Previous research has established a link between fibrinogen levels in sepsis patients and overall mortality, as well as associated organ dysfunction. Among individuals with septic shock, fibrinogen levels experience an average decline of 41% from day 1 to day 3, showing an inverse correlation with 30-day mortality risk (23). Furthermore, in the study on patients with SIC, the group exhibiting a declining trend in fibrinogen levels demonstrated the highest 28-day mortality rate compared to the group with a stable trend (46.7% *vs*. 10.0%, P=0.027), indicating a heightened risk of death (31). Similarly, our study showed the groups with intermediate and high fibrinogen levels displayed lower mortality rates compared with the low fibrinogen group(HR 0.67 *vs*. 0.81 *vs*. 1). Sepsis is characterized by inflammation and coagulation activation, with septic shock and coagulopathy signifying disease progression. During the inflammatory process, there is an increase in clotting factors and a decrease in fibrinolysis, resulting in fibrin deposition within the microcirculation, ultimately leading to thrombosis, microcirculatory disturbances, ischemia, and hypoxia, culminating in shock and organ dysfunction (32, 33). Moreover, excessive consumption of clotting factors leads to a significant reduction in fibrinogen and platelet count, contributing to adverse outcomes such as disseminated intravascular coagulation (DIC). Notably, the kidneys are particularly vulnerable to ischemia and hypoxia, with microcirculatory dysfunction potentially leading to SA-AKI (7, 34, 35). Therefore, the observed higher mortality rate among SA-AKI patients with low fibrinogen levels can be attributed to more severe vascular leakage, ischemia, and hypoxia. Additionally, in animal models of ischemia-reperfusion injury (36), the fibrinogen fragment Bβ15-42 has demonstrated protective effects by reducing inflammation and vascular leakage. Furthermore, in our study, the low fibrinogen group exhibited prolonged PTT and PT, as well as decreased platelet count, indicating an increased risk of bleeding and DIC, further emphasizing the association between low fibrinogen levels and adverse prognosis.

Contrary to previous findings associating high fibrinogen levels with cardiovascular diseases such as cardiac valve surgery and contrast-induced acute kidney injury (15, 17), our study reached the opposite conclusion. We discovered that SA-AKI patients with low fibrinogen levels have higher mortality rates, which may be related to the protective role of fibrinogen as an acute-phase protein in sepsis (28). Fundamental research has shown that fibrinogen protects cells from damage by binding to histones and fibrin released upon cell exposure (37). Therefore, increased fibrinogen concentration in the early stages of infection aids in preventing cell injury. However, during sepsis or trauma, when inflammation intensifies and immune function becomes excessively impaired, fibrinogen consumption exacerbates, diminishing its protective capacity. This leads to aggravated tissue injury, worsened prognosis, and increased mortality rates. Consistent with these observations, a study demonstrated that low fibrinogen levels remain a risk factor for in-hospital mortality in ICU AKI patients with sepsis (HR=1.29) (21). Our study yielded similar results. Moreover, our research revealed a nonlinear correlation between fibrinogen and SA-AKI, establishing a threshold of 1.6 g/l below which the risk of death significantly increases.

Patients with cirrhosis have an increased risk of developing AKI, which can eventually progress to hepatorenal syndrome and result in a significant risk of mortality (21). To account for potential interference from liver disease, we included liver disease as a confounder in the multivariate Cox analysis. Despite this adjustment, the association between fibrinogen and SA-AKI mortality remained robust. Subgroup analyses revealed that fibrinogen, as a continuous variable, exhibited a stronger association with 28-day mortality outcomes in patients with liver disease and SA-AKI than in those without liver disease (HR, 0.903;95% CI,0.825-0.990). As fibrinogen is produced by the liver, liver disease can result in reduced fibrinogen production and a poor prognosis for coagulopathy (38). Previous studies have demonstrated that low fibrinogen levels are an independent predictor of mortality in patients with acute-on-chronic hepatitis B liver failure (OR, 0.304; 95% CI: 0.094-0.983; *P* = 0.047) (39). Our subgroup results are consistent with these findings, indicating that fibrinogen levels are inversely associated with mortality risk in patients with liver disease, even in the context of SA-AKI. Patients undergoing RRT are at risk of bleeding from anticoagulant therapy (40). Fibrinogen is a predictor of the risk of bleeding prior to RRT. A reduction in fibrinogen levels is indicative of an elevated risk of bleeding and may portend a poor prognosis. As demonstrated in the subgroup analysis, fibrinogen exhibited a more pronounced inverse correlation in patients undergoing RRT compared to those not undergoing RRT (HR = 0.860, 95% CI: 0.757–0.977) (41). Although age is a risk factor for sepsis death, subgroup results show that compared with the elderly group ≥60 years, fibrinogen was more negatively associated with outcomes in younger than 60 years of age (HR: 0.861,95% CI: 0.788-0.940). This association warrants further investigation.

Nonetheless, this study has some limitations. Firstly, due to the limitations of the MIMIC database, the covariates (ALB, LAC) were excluded from the analysis owing to substantial missing data. However, a baseline comparison between groups with and without missing covariates was conducted, revealing minimal differences between the groups. Nevertheless, higher-quality prospective studies are needed in the future to validate our findings. Secondly, we only assessed fibrinogen levels within 24 hours after ICU admission without dynamic observation of patients’ fibrinogen levels, making it impossible to judge dynamic changes in fibrinogen for a comprehensive assessment of disease. Additionally, we did not consider the effect of fibrinogen infusion on outcomes. In the future, we will investigate the prognostic significance of changes in fibrinogen levels in patients with SA-AKI using the MIMIC database.

## 5 Conclusions

A non-linear association was observed between fibrinogen and SA-AKI. When fibrinogen was below 1.6 g/l, mortality risk decreased as fibrinogen increased.

## Data Availability

The original contributions presented in the study are included in the article/**Supplementary Material**. Further inquiries can be directed to the corresponding authors.
