# Supplementary material for: Prognostic significance of fibrinogen levels in sepsis-associated acute kidney injury: unveiling a nonlinear relationship and clinical implications
Source: Front Nephrol. 2024 Nov 6;4:1398386. doi: 10.3389/fneph.2024.1398386 (PMC11576428; doi:10.3389/fneph.2024.1398386)
Supplement: Supplementary file 1 [file Table1.docx]

**Table S1 GFR Category**

| **Category** | **Risk stratification** | **GFRml/(min×1.73m2)** | **n** |
| --- | --- | --- | --- |
| **GFR** |  | | |
| G 1 | low risk, | ≥90 | 23 |
| G 2 | low risk | 60-89 | 75 |
| G 3a | moderately increased risk | 45-59 | 112 |
| G 3b | high risk | 30-44 | 183 |
| G 4 | very high risk | 15-29 | 228 |
| G 5 | very high risk | ＜15 | 184 |
| **CKD** |  | | |
| No | - | ≥60 | 2674 |
| Yes | - | ＜60 | 805 |

GFR: glomerular filtration rate; CKD: Chronic Kidney Disease

low risk (if no other markers of kidney disease, no CKD)

**Table S2 Association between fibrinogen and SA-AKI in CKD (GFR) subgroup**

| Subgroup | event (%) | Unadjusted | | Adjusted | | *P* for interaction |
| --- | --- | --- | --- | --- | --- | --- |
|  |  | HR (95%CI) | *P* value | HR (95%CI) | *P* value |  |
| **GFR category** |  |  |  |  |  | 0.076 |
| G 1_2 | 15 (15.3) | 1.12 (0.87~1.44) | 0.381 * | 0.73 (0.3~1.82) | 0.505 |  |
| G 3_5 | 204 (28.9) | 0.96 (0.9~1.03) | 0.255 * | 0.97 (0.9~1.05) | 0.479 |  |
| **CKD** |  |  |  |  |  | 0.252 |
| No | 694 (26) | 0.934 (0.896~0.974) | 0.0014 * | 0.947 (0.902~0.993) | 0.0249 * |  |
| Yes | 219 (27.2) | 0.98 (0.919~1.045) | 0.533 * | 0.999 (0.927~1.077) | 0.9884 |  |

Note: *: *P* < 0.05; HR: hazard ratio.

GFR: glomerular filtration rate; CKD: Chronic Kidney Disease;

G 1_2**:** Including G 1 and G 2; G 3_5: Including G 3, G 4, G 5

Adjusted for sex, age、MAP、Respiratory-rate , HR、PLT, WBC, CR, PT, PTT, INR, ALB, LAC, MI, CHF, cerebrovascular-disease, COPD, DM, cancer, liver-disease, MST, SOFA, SAPS-III , CCI, MV, RRT, NE.

**Table S3 Sensitivity analysis of patients with liver disease**

| Variable | | unadjusted | | | Model 1 | | | Model 2 | | | Model 3 | |
| --- | --- | --- | --- | --- | --- | --- | --- | --- | --- | --- | --- | --- |
|  | HR (95%) | | *P* value | HR (95%) | | *P* value | HR (95%) | | *P* value | HR (95%) | | *P* value |
| fibrinogen(g/l) | 0.86 (0.8~0.93) | | <0.001* | 0.84 (0.77~0.91) | | <0.001* | 0.89 (0.82~0.98) | | 0.013* | 0.9 (0.82~0.99) | | 0.029* |
| fibrinogen(g/l) | | | | | | | | | | | | |
| Low (0.26-1.26) | 1(reference) | | _ | 1(reference) | | _ | 1(reference) | | _ | 1(reference) | | _ |
| Mid (1.27-2.09) | 0.57 (0.44~0.73) | | <0.001* | 0.53 (0.41~0.68) | | <0.001* | 0.65 (0.5~0.84) | | 0.001* | 0.75 (0.58~0.99) | | 0.042* |
| High(2.1-11.82) | 0.58 (0.46~0.75) | | <0.001* | 0.52 (0.41~0.67) | | <0.001* | 0.68 (0.51~0.92) | | 0.011* | 0.75 (0.55~1.03) | | 0.076 |
| *P* for trend | 0.75 (0.66~0.85) | | <0.001* | 0.71 (0.62~0.81) | | <0.001* | 0.81 (0.7~0.94) | | 0.006* | 0.86 (0.73~1) | | 0.057 |

Note: *: *P* < 0.05; HR: hazard ratio.

Model 1： sex, age

Model 2：sex, age, MAP, Respiratory-rate, HR, PLT, WBC, Cr, PT, PTT, INR, ALB, LAC.

Model 3：sex, age、MAP、Respiratory-rate , HR、PLT, WBC, CR, PT, PTT, INR, ALB, LAC, MI, CHF, cerebrovascular-disease, COPD, DM, CKD, cancer, MST, SOFA, SAPS-III , CCI, MV, RRT, NE.

**Table S4 Sensitivity analysis of patients with sepsis‐induced coagulopathy**

| Variable | | unadjusted | | | Model 1 | | | Model 2 | | | Model 3 | |
| --- | --- | --- | --- | --- | --- | --- | --- | --- | --- | --- | --- | --- |
|  | HR(95%) | | *P* value | HR(95%) | | *P* value | HR(95%) | | *P* value | HR(95%) | | *P* value |
| fibrinogen(g/l) | 0.95 (0.91~1) | | 0.036* | 0.94 (0.9~0.98) | | 0.009* | 0.93 (0.88~0.97) | | 0.003* | 0.97 (0.92~1.02) | | 0.214 |
| fibrinogen(g/l) | | | | | | | | | | | | |
| Low (0.26-1.68) | 1(reference) | | _ | 1(reference) | | _ | 1(reference) | | _ | 1(reference) | | _ |
| Mid(1.69-3.22) | 0.6 (0.5~0.72) | | <0.001* | 0.56 (0.47~0.68) | | <0.001* | 0.64 (0.53~0.78) | | <0.001* | 0.72 (0.59~0.88) | | 0.001* |
| High(3.23-14.66) | 0.72 (0.6~0.86) | | <0.001* | 0.66 (0.55~0.8) | | <0.001* | 0.63 (0.51~0.77) | | <0.001* | 0.77 (0.62~0.96) | | 0.019* |
| *P* for trend | 0.84 (0.76~0.92) | | <0.001* | 0.80 (0.73~0.89) | | <0.001* | 0.79 (0.71~0.87) | | <0.001* | 0.87 (0.78~0.97) | | 0.012* |

Note: *: *P* < 0.05; HR: hazard ratio.

Model 1： sex, age

Model 2：sex, age, MAP, Respiratory-rate, HR, PLT, WBC, Cr, PT, PTT, INR, ALB, LAC.

Model 3：sex, age、MAP、Respiratory-rate , HR、PLT, WBC, CR, PT, PTT, INR, ALB, LAC, MI, CHF, cerebrovascular-disease, COPD, DM, CKD, cancer, liver-disease, MST, SOFA, SAPS-III , CCI, MV, RRT, NE.

**Table S5. Non-missing group vs. missing group baseline information**

| Variables | Groups | | |
| --- | --- | --- | --- |
|  | Total | Non-missing group | Missing Group |
|  | (n = 6980) | (n = 3479) | (n = 3501) |
| **Baseline characteristics** | | | |
| Sex(Male),n (%) | 2657 (38.1) | 1381 (39.7) | 1276 (36.4) |
| Age(years) | 64.7 ± 14.8 | 62.5 ± 15.8 | 66.9 ± 13.4 |
| **Vital signs** |  |  |  |
| HR (bpm) | 87.7 ± 15.9 | 90.8 ± 17.5 | 84.8 ± 13.5 |
| MAP (mmHg) | 75.2 ± 9.0 | 75.8 ± 9.7 | 74.6 ± 8.3 |
| Respiratory-rate (bpm) | 19.4 ± 4.1 | 20.3 ± 4.5 | 18.5 ± 3.5 |
| **Laboratory examination** | | | |
| PLT(×109/L) | 126(86.0,176.0) | 119(72.0,179.0) | 130.5 (100.0,174.0) |
| fibrinogen(g/l) | 2.8 ± 1.8 | 2.9 ± 2.0 | 2.7 ± 1.6 |
| WBC(×109/L) | 9.7 (6.7, 13.2) | 9.5 (6.0, 13.5) | 9.8 (7.1, 12.9) |
| ALB(g/l) | 26 ± 6 | 26 ± 6 | 26 ± 6 |
| Cr(mg/dL) | 1.0 (0.7, 1.5) | 1.1 (0.8, 1.8) | 0.9 (0.7, 1.2) |
| INR | 1.4 ± 0.6 | 1.5 ± 0.7 | 1.3 ± 0.6 |
| PT(s) | 15.5 ± 6.5 | 16.3 ± 7.1 | 14.8 ± 5.7 |
| PTT(s) | 32.5 ± 11.2 | 33.5 ± 11.7 | 31.5 ± 10.6 |
| LAC (mmol/l) | 1.4 (1.1, 2.0) | 1.6 (1.1, 2.4) | 1.3 (1.0, 1.6) |
| **Disease severity score** | | | |
| SOFA | 4.0 (2.0, 6.0) | 4.0 (3.0, 6.0) | 3.0 (2.0, 5.0) |
| SAPS III | 62.1 ± 30.1 | 74.1 ± 30.3 | 50.3 ± 24.9 |
| **Comorbidities** | | | |
| MI (%) | 1446 (20.7) | 678 (19.5) | 768 (21.9) |
| CHF (%) | 2101 (30.1) | 1085 (31.2) | 1016 (29) |
| Cerebrovascular-disease (%) | 808 (11.6) | 433 (12.4) | 375 (10.7) |
| COPD (%) | 1713 (24.5) | 884 (25.4) | 829 (23.7) |
| DM (%) | 658 (9.4) | 311 (8.9) | 347 (9.9) |
| CKD (%) | 1522 (21.8) | 805 (23.1) | 717 (20.5) |
| Liver (%) | 1468 (21.0) | 1049 (30.2) | 419 (12) |
| Cancer (%) | 866 (12.4) | 528 (15.2) | 338 (9.7) |
| MST (%) | 294 (4.2) | 157 (4.5) | 137 (3.9) |
| CCI | 5.9 ± 2.8 | 6.0 ± 2.9 | 5.7 ± 2.6 |
| **Treatments using on day 1** | | | |
| MV (%) | 5953 (85.3) | 2900 (83.4) | 3053 (87.2) |
| RRT (%) | 535 (7.7) | 406 (11.7) | 129 (3.7) |
| NE (%) | 2254 (32.3) | 1595 (45.8) | 659 (18.8) |

Note: HR: Heart Rate; MAP: mean arterial pressure; PLT: platelet; WBC: white blood cell; ALB: albumin; Cr: creatinine; INR: international normalized ratio ; PTT: partial thromboplastin time; PT: prolonged prothrombin time; LAC: lactate; SOFA: sequential organ failure assessment; SAPS III :simplified acute physiology score III ;MI: myocardial infarct; CHF: congestive heart failure; COPD: chronic obstructive pulmonary disease; DM: diabetes; CKD: chronic kidney disease MST :metastatic solid tumor; CCI: charlson comorbidity index; MV :mechanic ventilation; RRT :renal replacement therapy ; NE :norepinephrine.
